# Harnessing Type II Cytokines to Reinvigorate Exhausted T Cells for Durable Cancer Immunotherapy

**DOI:** 10.1093/gpbjnl/qzae093

**Published:** 2024-12-26

**Authors:** Wenle Zhang, Yanwen Wang, Bin Li

**Affiliations:** Center for Immune-Related Diseases at Shanghai Institute of Immunology, Shanghai Jiao Tong University School of Medicine, Shanghai 200025, China; Center for Immune-Related Diseases at Shanghai Institute of Immunology, Shanghai Jiao Tong University School of Medicine, Shanghai 200025, China; Center for Immune-Related Diseases at Shanghai Institute of Immunology, Shanghai Jiao Tong University School of Medicine, Shanghai 200025, China

Cancer immunotherapy has made significant strides in developing immune checkpoint inhibitors and Chimeric Antigen Receptor (CAR)-based cellular therapies in recent years. Despite these advancements, the immunosuppressive nature of the tumor microenvironment (TME) and its metabolic complexity continue to present substantial challenges. Within this hostile environment, tumor-infiltrating T cells often experience severe functional impairment and reduced cytotoxic potential.

One of the main barriers to effective and long-lasting tumor control is T cell exhaustion — a state characterized by diminished effector functions, impaired proliferation, and sustained expression of inhibitory receptors like PD-1, TIM-3, and LAG-3. Although CAR-T cell therapy has revolutionized the treatment of hematological malignancies, relapse was constantly reported and limited efficacy was observed in treating solid tumors, largely due to the hostile TME and rapid onset of CAR-T cell exhaustion.

To better understand mechanisms behind sustained CAR-T cell responses, a large-scale single-cell multi-omics study profiled over one million pre-infusion CAR-T cells collected from 82 acute lymphoblastic leukemia patients, with clinical follow-up extending up to 10 years [1]. The analysis highlighted that elevated type II cytokine activity in CAR-T cell infusion products was linked to patients maintaining 8-year leukemia remission. At the chromatin level, higher increased accessibility was found at the locus of *GATA3*, while the *IFNG*, *STAT1*, *STAT4*, and *TBX21* loci showed negligible differences. Remarkably, the study also identified that this small population of type II cytokine-producing CAR-T cells could modulate dysfunctional subsets characterized by high expression of exhaustion markers and restricted proliferation. These findings challenge the traditional paradigm, which has primarily focused on type I immune responses (*e.g.*, Th1-driven responses) as the key drivers of effective tumor clearance.

Moreover, in a leukemia mouse model, type II-high CAR-T cells exhibited enhanced anti-tumor activities, especially upon tumor rechallenge. Priming CAR-T cells with IL-4 or incorporating IL-4 during CAR-T cell manufacturing improved their functional fitness and significantly prolonged tumor control.

In parallel, a long-lasting Fc–IL-4 fusion protein was engineered to significantly revitalize terminally exhausted CD8^+^ T cells through metabolic reprogramming in a lactate dehydrogenase A (LDHA)-dependent manner [2]. Moreover, Fc–IL-4 synergized with current type I immunity-centric adoptive T cell transfer and immune checkpoint blockade therapies to induce durable remission across several syngeneic and xenograft tumor models, which highlighted the potential of orchestrating type I and type Ⅱ immunity to advance next-generation cancer immunotherapy [2].

Similarly, CAR-T cells expressing IL-10, another type II cytokine traditionally associated with immune suppression, exhibited better control of solid tumors, highlighting the pivotal role of IL-10 in reversing T cell dysfunction [3]. This is a departure from IL-10’s conventional role as an immunosuppressive cytokine secreted by regulatory T cells (Tregs), which promotes immune tolerance and contributes to a tumor-promoting microenvironment [4]. In contrast, IL-10 expressed by CD8^+^ T cells promoted the proliferation, effector function, and longevity of T cells which mediated durable clearance of solid tumors, suggesting that the source and context of IL-10 expression are critical in determining its role [5].

Intriguingly, the metabolic reprogramming on terminally exhausted T cells driven by IL-4 and IL-10 was different. IL-4 mainly augmented the glycolytic metabolism through both PI3K-AKT-mTOR and STAT6 signaling [2]. In contrast, IL-10 could also sustain mitochondrial fitness and OXPHOS metabolism through STAT3 signaling alone [5]. More importantly, terminally exhausted T cells could directly respond to the type Ⅱ cytokines and obtain enhanced proliferative capacity and cytotoxic potential (**Figure 1**). This indicates that terminally exhausted T cells, typically resistant to PD-1 inhibitors, might still be amenable to reprogramming via cytokine or metabolic modulation. The type Ⅱ cytokines could synergize with immune checkpoint inhibitors (*e.g.*, anti-PD-1 blocking antibody) to expand the CAR-T therapy beyond hematological malignancies into solid tumors. This redefinition of T cell exhaustion heterogeneity, based on cytokine-receptor expression profiles, could pave the way for novel therapeutic strategies.

Traditionally, type II cytokines are considered detrimental in cancer therapy due to their association with immunosuppressive responses, such as M2 macrophages and Tregs, which promote tumor growth. Nonetheless, under specific conditions, type II cytokines can synergize with type I responses to restore immune competence. This insight calls for a re-evaluation of how type II cytokines are utilized in cancer treatment, emphasizing their potential to optimize immune responses when paired with type I cytokines like IFN-γ. Optimizing the conditions for leveraging type II cytokines and integrating these strategies with established therapies, such as PD-1 blockade and standard CAR-T protocols, remain an area for further exploration. Additionally, advances in single-cell profiling technology could play a key role in tailoring CAR-T cell products by identifying the optimal cytokine signatures and metabolic states associated with long-term remission and improved clinical outcomes.

These findings suggest a significant paradigm shift in cancer immunotherapy, highlighting the role of type II cytokines not only in enhancing cytotoxicity but also in maintaining balanced T cell functionality, adaptability, and metabolic health [6]. This shift is especially pertinent in the context of CAR-T cell therapies, where overactivation can drive rapid exhaustion and diminished efficacy. Recent studies further highlight that T cells, as ‘living drugs’, can be finely tuned to adapt dynamically to the niches and establish durable remission in refractory autoimmune diseases by re-establishing immune homeostasis [7]. Even in immunosuppressive FOXP3^+^ Tregs, targeting the perturbation of the IFN-γ-STAT1-IFITM3 pathway can reprogram them to potentiate the anti-tumor responses [8]. Thus, maintaining T cell resilience and strategically leveraging cellular perturbation could yield durable and systemic therapeutic effects for both malignancies and autoimmune diseases [9].

Future strategies may focus on engineering cells with bispecific cytokine receptors or synthetic circuits to deliver balanced signals for both short-term cytotoxic activity and long-term persistence. Developing bispecific cytokines that simultaneously engage type I and type II pathways represents a promising approach. Furthermore, the combination of different cytokine profiles may contribute to optimizing CAR-T cell therapy for enhanced infiltration, cytotoxicity, and longevity. For example, combining type I and type II cytokines with factors like IL-7 and CCL-19 could potentially achieve improved infiltration and killing capacity in solid tumors [10].

Overall, these findings highlight a new avenue to enhance immunotherapy by reprogramming T cell metabolism and modulating the TME through the strategic use of cytokines. This approach could reshape how CAR-T cells are engineered and utilized, potentially expanding their applicability and success across a broader spectrum of cancer types. Moreover, the success of CAR-T cell therapy serves as a model for other cell-based treatments, reinforcing the potential of cytokine modulation and autocrine signaling as universal approaches to optimize T-cell performance and improve therapeutic outcomes.

## CRediT author statement

**Wenle Zhang:** Conceptualization, Writing – original draft, Writing – review & editing. **Yanwen Wang:** Conceptualization, Writing – original draft, Visualization. **Bin Li:** Conceptualization, Writing – review & editing, Funding acquisition, Supervision. All authors have read and approved the final manuscript.

## Competing interests

The authors have declared no competing interests.

## Figures and Tables

**Figure 1:**
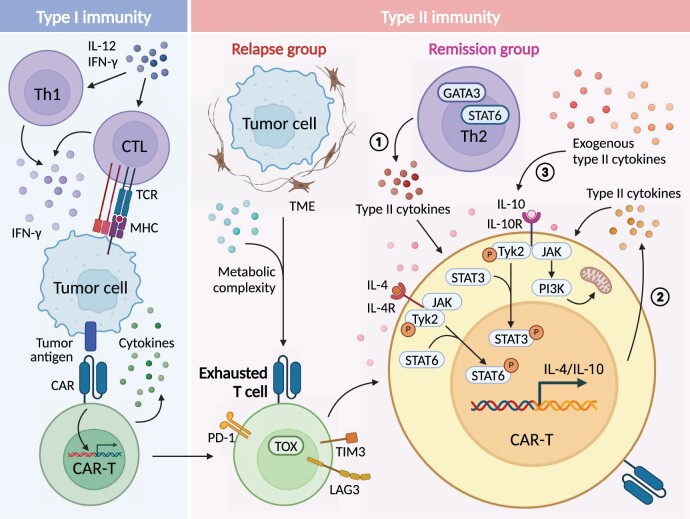
Schematic overview of type II immunity enhancing CAR-T resilience and anti-tumor effects Type I immunity (left) drives tumor cytotoxicity through Th1 and CTLs but leads to CAR-T exhaustion, characterized by increased expression of inhibitory receptors such as PD-1, TIM3, and LAG3. Type II immunity (right) enhances CAR-T cell resilience through IL-4 and IL-10 signaling via the JAK/STAT pathways, thereby improving metabolic fitness, anti-tumor function, and long-term persistence. The sources of type II cytokines can be primarily categorized into three aspects: (1) cytokines secreted by type II immune cells in the environment; (2) type II cytokines self-secreted by genetically modified CAR-T cells; (3) exogenous type II cytokines, including those used for pre-treatment before infusion and those in the form of Fc fusion proteins. The image was created with BioRender.com. CAR, chimeric antigen receptor; Th1, T helper 1; CTL, cytotoxic T lymphocyte; TCR, T cell receptor; MHC, major histocompatibility complex; TME, tumor microenvironment; Th2, T helper 2; PD-1, programmed cell death protein 1; TOX, thymocyte selection associated high mobility group box; TIM3, T-cell immunoglobulin and mucin-domain containing-3; LAG3, lymphocyte-activation gene 3; JAK, Janus kinase; STAT, signal transducer and activator of transcription; PI3K, phosphoinositide 3-kinase; GATA3, GATA-binding protein 3; Tyk2, tyrosine kinase 2; IL-4, interleukin-4; IL-10, interleukin-10; IL-12, interleukin-12; IL-4R, interleukin-4 receptor; IL-10R, interleukin-10 receptor; IFN-γ, interferon-gamma.
